# METTL14 promotes prostate tumorigenesis by inhibiting THBS1 via an m6A-YTHDF2-dependent mechanism

**DOI:** 10.1038/s41420-022-00939-0

**Published:** 2022-03-30

**Authors:** Yongjie Wang, Junfei Chen, Wei-Qiang Gao, Ru Yang

**Affiliations:** 1 State Key Laboratory of Oncogenes and Related Genes, Renji-MedX Clinical Stem Cell Research Center, Ren Ji Hospital, School of Medicine, Shanghai Jiao Tong University, 200127 Shanghai, China; 2 State Key Laboratory of Oncogenes and Related Genes, Shanghai Cancer Institute, Renji Hospital, School of Medicine, Shanghai Jiao Tong University, 200032 Shanghai, China; 3 School of Biomedical Engineering and Med-X Research Institute, Shanghai Jiao Tong University, 200030 Shanghai, China

**Keywords:** Gene silencing, Methylation, Prostate cancer, Tumour biomarkers

## Abstract

N6-methyladenine (m6A) is the most predominant RNA modification, which has been shown to be related to many types of cancers. However, understanding of its role in prostate cancer (PCa) is largely unknown. Here, we report an upregulation of METTL14 that was correlated with poor prognosis in PCa patients. Functionally, knocking down METTL14 inhibited tumor proliferation both in vitro and in vivo. Mechanically, RNA-seq and MeRIP-seq analyses identified THBS1 as the downstream target of METTL14 in PCa. METTL14 downregulated THBS1 expression in an m6A-dependent manner, which resulted in the recruitment of YTHDF2 to recognize and degrade Thrombospondin 1 (THBS1) mRNA. Thus, our findings revealed that METTL14 acted as an oncogene by inhibiting THBS1 expression via an m6A-YTHDF2-dependent manner. METTL14 could be a potential prognosis marker and a therapeutic target.

## Introduction

Prostate cancer (PCa) is the most common male cancer and represents the sixth cause of cancer death in men worldwide [1, 2]. Nowadays, for patients with advanced PCa, androgen deprivation therapy (ADT) has been the primary therapy for decades of years. Despite the initial response to androgen deprivation therapy (ADT), the majority of patients relapse with a poor prognosis stage of castration-resistant prostate cancer (CRPC). Patients with CRPC cannot be cured currently and the mortality remains high [3, 4]. Thus, understanding of the mechanisms underlying PCa progression is essential for molecular diagnosis and targeted therapy.

Traditionally, epigenetic regulations consist of diverse modifications on DNA and histone, which are a series of reversible biological processes regulating gene expression without changing the genome sequences [5]. Previous studies have found that many epigenetic regulators on DNA and histone methylation have important effects on PCa progression [6]. These studies have provided a novel epigenetic view for exploring promising targeted therapies for PCa. Apart from these regulators on DNA or histone, in recent years, accumulating studies have focused on RNA modification, especially methyladenine (m6A) modification, which is the most prevalent post-transcriptional alteration [7, 8]. As a dynamic and reversible process, m6A modification is installed to RNA by m6A methyltransferases(writers), including methyltransferase-like 3 (METTL3) [9], methyltransferase-like14 (METTL14) [10], and Wilms tumor 1 associated protein (WTAP) [11], and removed by alkylation repair homolog protein(ALKBH5) and Fat mass and obesity-associated protein (FTO), which both act as m6A demethylases(erasers) [12]. Besides, there are some proteins, called m6A readers, exerting their functions in recruiting and binding to m6A sites, like YTH domain-containing family protein 1/2/3(YTHDF1/2/3) and insulin-like growth factor 2 mRNA binding proteins 1/2/3(IGF2BP1/2/3) [12]. The alterations of these m6A effectors have been implicated in many types of cancers, such as lung cancer [13], hepatocarcinoma [14], and colorectal cancer [15]. It has been previously shown that METTL3 drives migratory and invasive capacities of PCa cells via mediating m6A modification of USP4 mRNA in a YTHDF2-dependent manner [16]. METTL14, as the indispensable allosteric activator of METTL3, has been shown to play an important role in tumor progression in many types of cancers [17–19]. However, the biological significance of METTL14 in PCa has not been elucidated.

In this study, we report that METTL14 promoted the progression of PCa and identified Thrombospondin 1 (THBS1), an endogenous inhibitor of angiogenesis [20], as its downstream target. Moreover, a member of m6A readers, YTHDF2, was recruited for THBS1 mRNA decay. Collectively, our investigation proposes that METTL14 may be a novel prognosis marker and a potential therapeutic target for PCa.

## Results

### Upregulation of METTL14 is correlated with poor prognosis of PCa patients

**Fig. 1:**
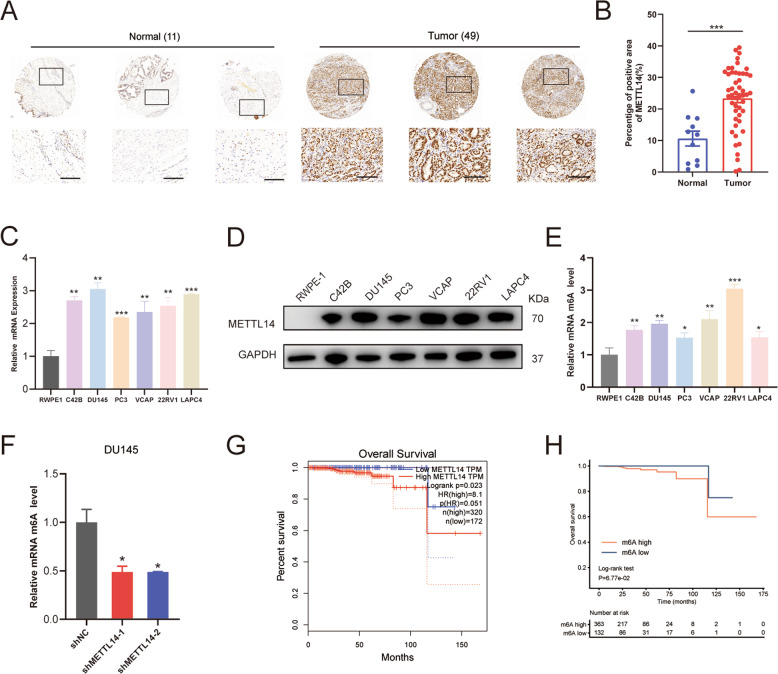
Upregulation of METTL14 is correlated with poor prognosis of PCa patients. **A** Representative images of IHC staining of PCa tumor tissues (*n* = 49) and normal ones (*n* = 11) with the anti-METTL14 antibody. up: scale bar: 400 μm, down: scale bar: 100 μm. **B** The calculation of percentage of positive area of METTL14 in PCa tumor tissues (*n* = 49) and normal ones (*n* = 11) for TMA. **C, D** The relative mRNA (**C**) and protein (**D**) levels of METTL14 in RWPE1 and PCa cell lines detected by qPCR and western. **E** The relative m6A quantification of RWPE1 and PCa tumor cell lines. **F** Two independent shRNA sequences targeting METTL14 (shMETTL14-1 and shMETTL14-2) were separately transfected into DU145 cells. The relative m6A quantification of control and METTL14 knockdown in DU145 cells. **G** Overall survival of PCa patients with high and low METTL14 mRNA level using Kaplan–Meier survival curve analysis methods based on the TCGA dataset. **H** Overall survival of PCa patients with high and low m6A expression classified by consensus clustering. The data in **B** is presented as the mean ± SEMs. The other data are presented as the mean ± SDs. *p < 0.05; **p < 0.01; ***p < 0.001 (Student’s *t*-test).

To quantify METTL14 expression in PCa patients, we performed IHC staining on a tissue microarray containing prostate tumor tissue specimens (*n* = 49) and adjacent normal prostate tissue specimens (*n* = 11). The staining results indicated that METTL14 was highly expressed in PCa tissues compared to the normal ones (Fig. 1A, B). Similarly, the higher expression levels of METTL14 mRNA (Fig. 1C) and protein (Fig. 1D) were observed in human PCa cell lines compared with the normal prostate cell line RWPE1, a prostatic epithelial cell line. Besides, the relative m6A quantifications of RWPE1 and PCa tumor cell lines were measured. The results revealed that PCa tumor cell lines exhibited higher m6A levels than RWPE1 (Fig. 1E). Furthermore, DU145 and PC3 are two classical CRPC cell line models among the cell lines used above [21]. Due to that DU145 presents a higher m6A level than PC3, m6A quantification assays were conducted on DU145 and we found that knocking down METTL14 decreased the m6A level in DU145 (Fig. 1F).

In addition, those PCa patients with a higher METTL14 expression exhibited a shorter overall survival (OS) than those with a lower METTL14 expression (Fig. 1G). Moreover, to investigate the association between m6A level and the prognosis of PCa patients, we analyzed the PCa TCGA (The Cancer Genome Atlas) dataset and found that the patients with a high m6A level showed a worse OS compared to those with a low m6A level (Fig. 1H, Supplementary Fig. 1A), which indicated that the high m6A level was an adverse prognostic factor. Taken together, these results indicate that the upregulated METTL14 expression influences the prognosis of PCa patients via mediating the m6A modification.

### METTL14 promotes PCa cell proliferation in vitro and in vivo

**Fig. 2:**
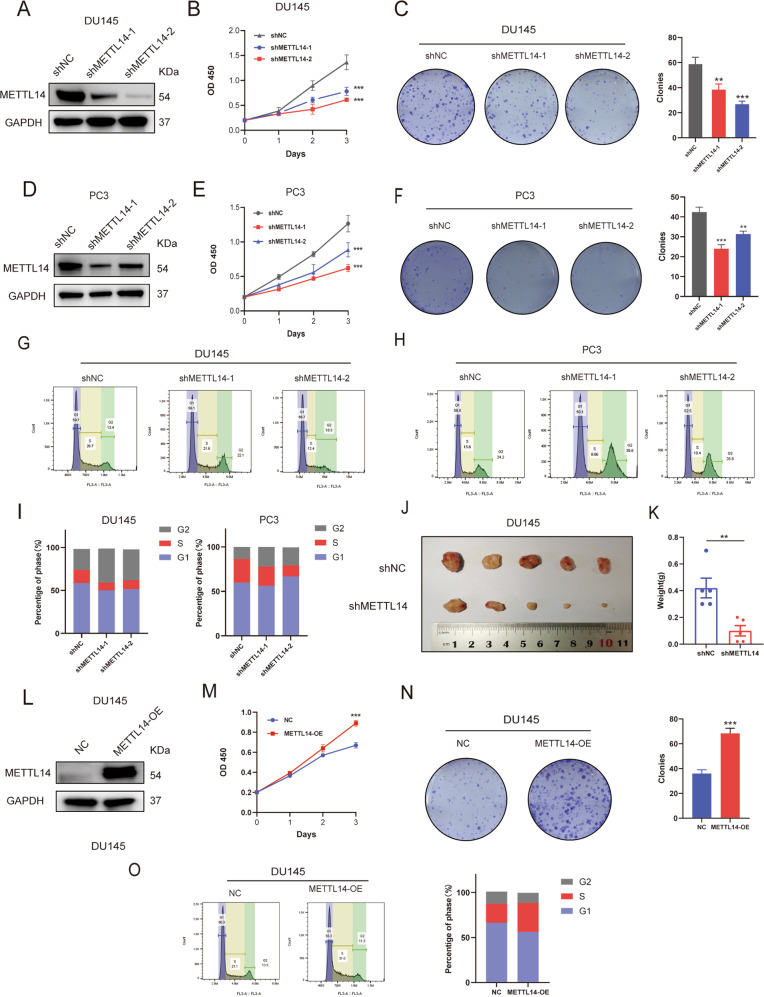
METTL14 promotes PCa cell proliferation in vitro and in vivo. **A, D** Two independent shRNA sequences targeting METTL14 (shMETTL14-1 and shMETTL14-2) were separately transfected into DU145 or PC3 cells. Knocking down efficiency of METTL14 in DU145 and PC3 cells was confirmed by western blot. **B, E** Cell proliferation assays for control and METTL14 knockdown DU145 (**B**) and PC3 (**E**) cells were conducted. **C, F** Colony formation assays were performed and the colony numbers in DU145 (**C**) and PC3 (**F**) cells were measured. **G–I** Cell cycle assays (**G, H**) were performed and the percentages of each phase (**I**) in DU145 and PC3 cells were calculated. **J** The control and METTL14 knockdown DU145 cells were subcutaneously injected into the nude mice (*n* = 5). **K** Tumor weight of each group was measured. **L** Overexpression constructs of METTL14 (METTL14-OE) were stably transfected into DU145 and PC3 cells, respectively. Overexpression efficiency of METTL14 in DU145 cells was confirmed by western blot. **M–O** Cell proliferation assays (**M**), colony formation assays (**N**), and cell cycle assays (**O**) for control and METTL14 overexpression cells were conducted. The data in **K** is presented as the mean ± SEMs. The other data are presented as the mean ± SDs. *p < 0.05; **p < 0.01; ***p < 0.001 (Student’s *t*-test).

To explore the role of METTL14 in PCa, we established the stable METTL14 knockdown and overexpression cell lines based on CRPC cell lines DU145 (Fig. 2A, L, Supplementary Fig. 1B) and PC3 (Fig. 2D, Supplementary Fig. 1C). We found that depleting METTL14 reduced the cell proliferation and the colony formation efficiency remarkably in DU145 (Fig. 2B, C) and PC3 (Fig. 2E, F) cells. On the contrary, overexpression of METTL14 amplified the proliferation and colony numbers of tumor cells (Fig. 2M, N). Furthermore, cell cycle assays were carried out to further confirm the function of METTL14 for cell proliferation. The results showed that the proportion of cells in the G2 phase was increased and the proportion of cells in the S phase was decreased in METTL14 knockdown cell lines (Fig. 2G-I), whereas, elevated expression of METTL14 effectively decreased the proportion of the G2 phase and increased the proportion of cells in S phase (Fig. 2O).

In addition, to validate the function of METTL14 for tumor growth in vivo, we subcutaneously injected stably transfected METTL14 knockdown DU145 cell lines in nude mice. Then after 6 weeks, the data showed that the depletion of METTL14 significantly restricted the tumor growth. Compared to the control group, the shMETTL14 group exhibited smaller tumor volumes and weights (Fig. 2J, K).

These data together suggest that METTL14 results in accelerated cell proliferation in PCa.

### Identification of METTL14 targets via RNA-seq and MeRIP-seq

**Fig. 3:**
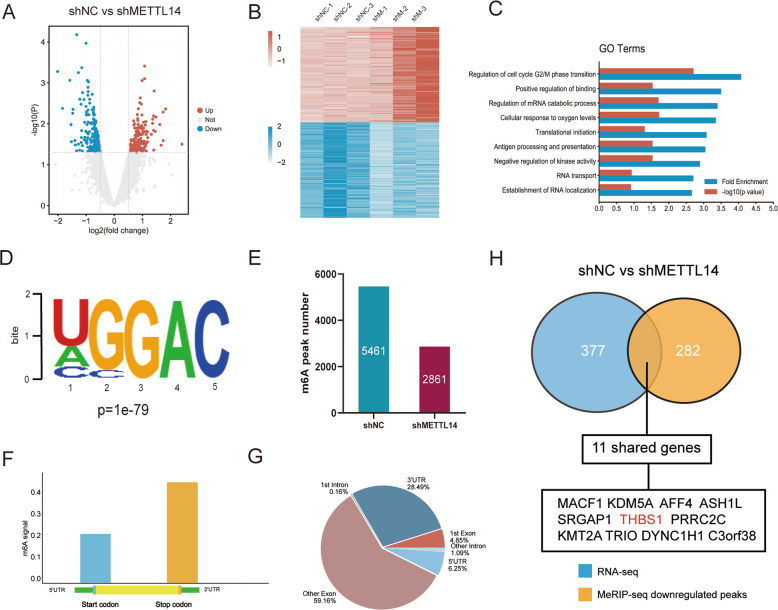
Identification of METTL14 targets via RNA-seq and MeRIP-seq. **A** Volcano plot of DEGs identified by RNA-seq in shNC and shMETTL14 DU145 cells. Gray, unchanged genes (|log2(fold change) | < 0.5 or *p* > 0.05) when comparing shNC and shMETTL14 group. Red, upregulated genes (log2(fold change) > 0.5 and *p* < 0.05) in shMETTL14 group. Blue, downregulated genes (log2(fold change) < −0.5 and *p* < 0.05). **B** Heatmap of upregulated and downregulated DEGs. **C** GO analysis of DEGs. **D** The consensus motif of DU145 cells identified by MeRIP-seq. *p* = 1e-79. **E** The number of m6A peaks in shNC (*n* = 5461) and shMETTL14 (*n* = 2861) group. **F** The m6A signals were largely enriched in 3’UTR. **G** Distribution of peaks in DU145 cells **H** Determination of shared genes of RNA-seq and MeRIP-seq. 11 genes were measured. THBS1 was identified as the potential target of METTL14.

To further investigate the mechanism underlying how METTL14 promotes tumor progression and identify its downstream targets in PCa, we conducted RNA-sequencing (RNA-seq) and Methylated RNA immunoprecipitation sequencing (MeRIP-seq) using shNC and shMETTL14 DU145 cells. RNA-seq analysis showed that compared to the control group, 204 genes (log2 FC < −0.5) were downregulated, and 172 genes (log2 FC > 0.5) were upregulated when METTL14 was knocked down in tumor cells (Fig. 3A, B, Supplementary Table 3). Differential genes were found to be linked with regulation of mRNA stability, cell cycle G2/M phase transition, positive regulation of binding, and RNA transport using Gene Ontology (GO) analysis (Fig. 3C, Supplementary Table 4).

For MeRIP-seq, consistent with previous studies [22], GGAC was the most frequent m6A motif enriched in our detected peaks (Fig. 3D). In total, MeRIP-seq analysis identified 5461 and 2861 peaks in shNC and shMETTL14 cells, respectively (Fig. 3E). Besides, the m6A signal was largely enriched in 3’ UTR (Fig. 3F, G). To assess whether the changed gene expression was induced by m6A modification, we then overlapped 282 reduced m6A peaks of MeRIP-seq (Supplementary Table 5) and 377 altered genes of RNA-seq, and found that 11 genes were both detected (Fig. 3H). Among these genes, we found that THBS1, an endogenous inhibitor of angiogenesis [20], presented the most consistent decreased m6A level, and its mRNA level became increased in shMETTL14 PCa cells compared to control cells (Supplementary Fig. 1E, F), we therefore chose THBS1 as a potential targeted gene after METTL14 m6A modification for further studies.

### THBS1 is regulated by METTL14-mediated m6A modification

**Fig. 4:**
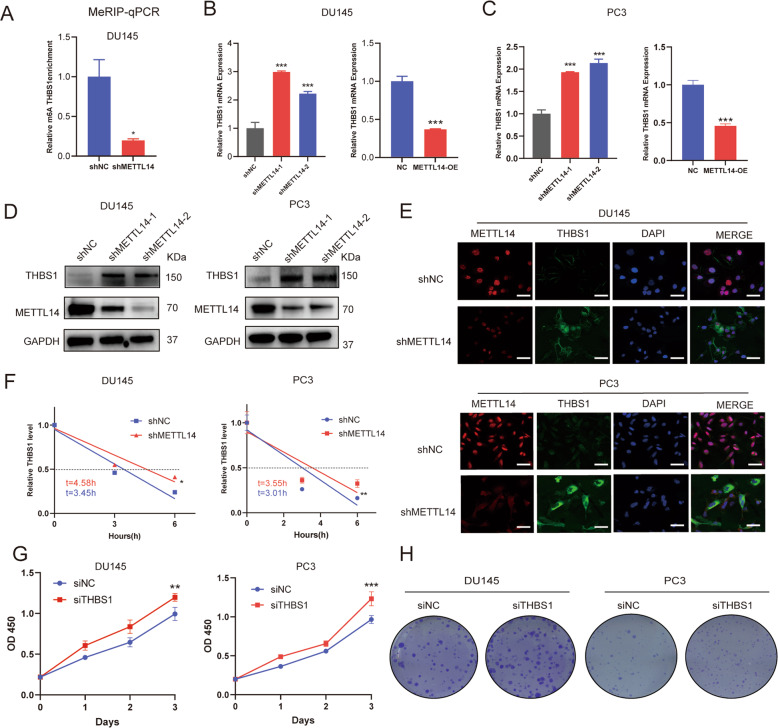
THBS1 is regulated by METTL14-mediated m6A modification and acts as a potential tumor suppressor in PCa cells. **A** The relative m6A enrichment of THBS1 determined by MeRIP-qPCR. **B, C** The mRNA level of THBS1 when knocking down (left) or overexpressing (right) METTL14 in DU145 (**B**) and PC3 (**C**) cells. **D** The protein levels of THBS1 after METTL14 deletion in DU145 and PC3 cells. **E** The expression and location of METTL14 and THBS1 measured by Immunofluorescence (IF) assays in DU145 or PC3 shNC and shMETTL14 cells. Scale bar: 20 μm. **F** The decay rate of THBS1 mRNA determined at 0, 3, 6 h after treating with actinomycin D (5ug/ml) in METTL14 knocking down and METTL14 overexpression DU145 cells. **G, H** The siRNA sequences targeting THBS1 (siTHBS1) were transfected into DU145 or PC3 cells. Cell proliferation assays (**G**) and colony formation assays (**H**) were conducted. All data are presented as the mean ± SDs. *p < 0.05; **p < 0.01; ***p < 0.001 (Student’s *t*-test).

To validate whether THBS1 is a target of METTL14-mediated m6A modification, as identified in MeRIP-seq, we performed MeRIP-qPCR in PCa cells. The m6A enrichment of THBS1 was remarkably decreased when METTL14 was knocked down (Fig. 4A). Furthermore, THBS1 mRNA level was increased in PCa METTL14 knockdown cells and decreased in PCa overexpression cells (Fig. 4B, C). Moreover, the protein level of THBS1 was also increased when METTL14 was knocked down (Fig. 4D). We also found that the METTL14 protein was located in the nucleus while THBS1 protein was located in the cytoplasm by Immunofluorescence (IF) assays. METTL14 expression exhibited a rise in PCa cells after METTL14 was knocked down (Fig. 4E). In addition, the half-life of THBS1 RNA was extended in METTL14 knockdown cells after the cultures were treated with actinomycin D, which is a transcriptional inhibitor (Fig. 4F). Taken together, our results reveal that METTL14 mediates mRNA degradation via an m6A-dependant manner to inhibit THBS1 expression.

### THBS1 is a potential tumor suppressor in PCa

To investigate the role of THBS1 in PCa progression, cell proliferation assays were conducted and the data showed that after knocking down THBS1 using targeted siRNA (Supplementary Fig. 1D), the growth of PCa cells was increased (Fig. 4G). The same trend was also observed in colony formation assays, we found that knocking down THBS1 significantly facilitated tumor cells colony formation ability (Fig. 4H). Collectively, these results point out that THBS1 may act as a potential tumor suppressor to suppress PCa proliferation.

### YTHDF2 facilitates THBS1 mRNA decay in an m6A-dependent manner

**Fig. 5:**
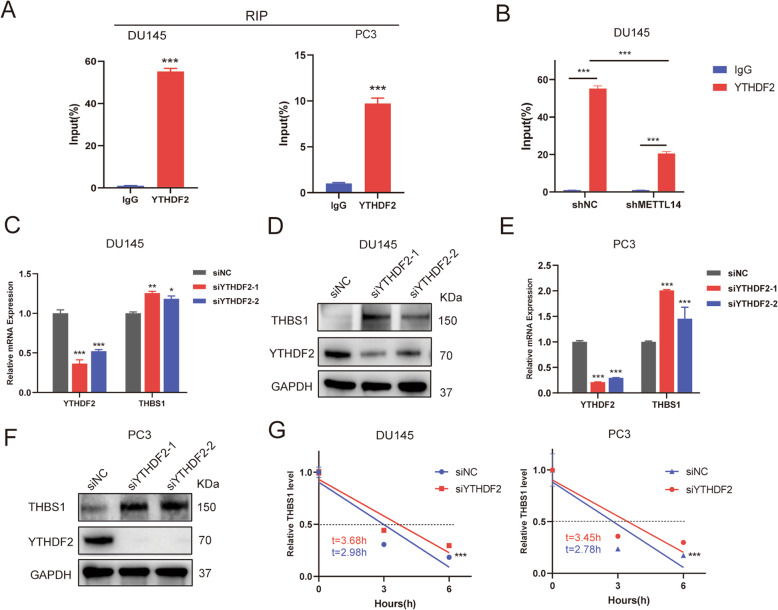
YTHDF2 facilitates THBS1 mRNA decay in an m6A-dependent manner. **A** RIP assays of DU145 and PC3 cells followed by qPCR showing the direct binding of YTHDF2 and THBS1. **B** RIP-qPCR exhibiting the enrichment of THBS1 in DU145 cells after inhibiting METTL14. **C, D** Two independent siRNA sequences targeting YTHDF2 (siYTHDF2-1 and siYTHDF2-2) were separately transfected into DU145 or PC3 cells. The YTHDF2 and THBS1 mRNA (**C**) and protein (**D**) levels after inhibiting YTHDF2 in DU145 cells. **E, F** The YTHDF2 and THBS1 mRNA (**E**) and protein (**F**) level after inhibiting YTHDF2 in PC3 cells. **G** The decay rate of THBS1 mRNA when inhibiting YTHDF2 using targeted siRNAs at 0, 3, 6 h after actinomycin D (5ug/ml). All data are presented as the mean ± SDs. *p < 0.05; **p < 0.01; ***p < 0.001 (Student’s *t*-test).

The m6A methylation added by m6A writers needs to be recognized by m6A readers for increasing or decreasing gene expression [23, 24]. Among them YTHDF2 is known to be the regulator of promoting mRNA degradation [25], which may be recruited by METTL14 to mediate THBS1 mRNA decay. To test this conjecture, we firstly conducted RNA immunoprecipitation (RIP) assays to assess whether YTHDF2 can directly bind to THBS1 mRNA. The RIP analyses showed that the groups with antibodies against YTHDF2 bound much more THBS1 mRNA than those against IgG in DU145 and PC3 cells (Fig. 5A). Besides, the binding between YTHDF2 and THBS1 was weakened when METTL14 was inhibited (Fig. 5B). To further examine the effect of YTHDF2 binding to THBS1 mRNA on their expression levels, we knocked down YTHDF2 with two targeted siRNA and found that both mRNA and protein expression of THBS1 was elevated (Fig. 5C-F). Furthermore, the RNA stability assays revealed that after treatment with siRNA targeted YTHDF2, the decay rate of THBS1 exhibited a significantly slower trend (Fig. 5G). These data demonstrate that YTHDF2 recognizes METTL14-methylated THBS1 mRNA and accelerates THBS1 mRNA decay.

## Discussion

**Fig. 6:**
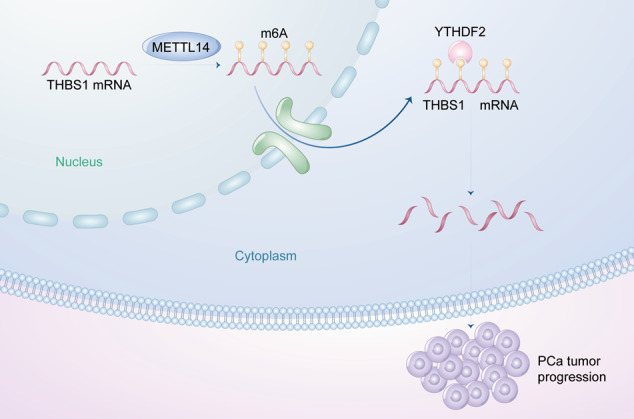
The graphic illustration of this study. METTL14 exerts m6A modification on THBS1 mRNA via directly binding to its 3’UTR region in nucleus. Then the m6A modified THBS1 mRNA is recognized by YTHDF2 in cytoplasm and degraded by YTHDF2. Taken together, the METTL14-YTHDF2-THBS1 axis promotes PCa progression.

Prostate cancer (PCa) has the highest incidence among all cancers in men and the treatment options for CRPC are limited [1, 2]. Nowadays, looking for new mechanisms underlying PCa progression and finding novel therapeutic targets are urgently needed. Recent research has demonstrated that the abnormal gene expression of m6A regulators plays a significant role in PCa progression. In our study, we for the first time demonstrate that elevated METTL14, a key m6A writer, is involved in the worse prognosis of PCa patients. Functionally, we find that METTL14 could promote the proliferation of PCa both in vitro and in vivo. Mechanistically, our GO analysis of RNA-seq related to the association between regulation of mRNA stability, positive regulation of binding and RNA transport, and RNA modifications validate our conjecture that METTL14 promotes PCa proliferation through RNA modification. In line with other studies [26], our MeRIP-seq sequencing results show that the m6A motif of DU145 cells is located around 3’UTR. Furthermore, by integrating RNA-seq and MeRIP-seq differential genes, we have identified their shared gene THBS1 as the target gene of METTL14. METTL14 promotes tumor progression by binding to THBS1 3’ UTR region and decreasing its expression. Moreover, methylated THBS1 mRNA is recognized by m6A reader YTHDF2. YTHDF2 further binds to THBS1 mRNA and accelerates THBS1 mRNA decay (Fig. 6). Overall, all these studies provide a novel epigenetic dimension for exploring the pathogenesis of PCa. METTL14 could be a potential prognosis marker and a therapeutic target for PCa in the future.

The m6A modification exerts its functions through three types of proteins, called writer, eraser, and reader, respectively. Via its dynamic methylation and demethylation, m6A modification has been proven to be involved in the pathological processes of many types of cancers [27, 28]. In prostate cancer, the m6A writer METTL3 has been confirmed to promote the proliferation and metastasis of PCa [16]. It is also found that one of the m6A readers, YTHDF2, accelerates PCa progression by mediating the mRNA degradation of the tumor suppressors [29]. However, functioning as the key methyltransferase, the role of METTL14 in PCa is still unknown. Here, we for the first time illustrate that METTL14 serves as the oncogene in the pathological processes of PCa and accelerates tumor proliferation in PCa. Considering that the metastasis ability of tumor cells is also a crucial factor for both tumor progression and patients’ prognosis, further studies will be performed to explore how METTL14 affects the metastasis ability of PCa tumor cells in the future.

Based on our work, we further identified THBS1 as the potential target gene of METTL14 and functions as the tumor suppressor in PCa. METTL14 inhibits THBS1 expression in an m6A-dependent manner to promote PCa proliferation. THBS1 is a glycoprotein that is a natural inhibitor of angiogenesis [30]. Neoplastic growth has been shown to be dependent on vessels to supply nutrients and oxygen. Thus, inhibition of angiogenesis can be an effective way to restrict tumor proliferation [31]. Lower THBS1 expression is reported to be associated with many kinds of cancers [20, 32], indicating that THBS1 can serve as a tumor suppressor. Notably, in neuroendocrine prostate cancers (NEPC) [33], THBS1 is identified as the downstream target of tri-methylating lysine 27 of histone H3 (H3K27me3) enzyme EZH2, and its lower expression was associated with NEPC tumorigenesis. Thus, these results together support the notion that THBS1 can be a tumor suppressor in NEPC. In our study, apart from the modification of histone modification above, we also discovered another epigenetic modulation on THBS1. First, we demonstrated that THBS1 expression is inhibited by METTL14 in an m6A-dependent manner in the nucleus. Second, we have identified the biological function of THBS1 as a potential target to suppress tumor growth. Third, methylated THBS1 mRNA can be recognized by m6A reader YTHDF2. Among m6A readers, YTHDF2 functions as promoting mRNA decay by binding to m6A sites in the cytoplasm [34]. Moreover, the sequencing results show that METTL14 binds to THBS1 3’ UTR region, so the specific binding site needs to be further investigated by luciferase reporter assay and single-nucleotide m6A detection methods. Additionally, THBS1, as a tumor suppressor inhibiting PCa proliferation, can be considered as the potential therapeutic target in the future.

Taken together, our study reveals an oncogenic role of METTL14 in PCa progression. Mechanistically, we identified a “METTL14-YTHDF2-THBS1” axis in PCa cells, which provides a novel epigenetic dimension for exploring the pathogenesis of PCa. Thus, METTL14 could be a potential prognosis marker and a therapeutic target for PCa.

## Materials and methods

### Cell culture and reagents

All cell lines were obtained from the American Type Culture Collection (ATCC). Human prostate cancer cell lines DU145 were cultured in DMEM medium (Gibco, Grand Island, NY), while PC3 was cultured in RPMI-1640 medium (Gibco, Grand Island, NY). Both mediums were supplemented with 10% fetal bovine serum (FBS). Cells were grown at 37 °C in a cell culture incubator containing 5% CO_2_.

### TCGA dataset analysis

RNA-sequencing (RNA-seq) data of TCGA-PRAD cohort were achieved through the Cancer Genome Atlas portal (https://portal.gdc.cancer.gov/). Fragments Per Kilobase of transcript per Million mapped reads (FPKM) was transformed into Transcripts Per Million (TPM) values for subsequent analysis. The survival data of TCGA-PRAD cohort was obtained from the TCGA Pan-Cancer Clinical Data Resource (TCGA-CDR) [35]. We divided samples into METTL14 high and low groups by median TPM of METTL14. Consensus Clustering was performed based on gene expressions of m6A and identified m6A low and high groups.

### Tissue microarray (TMA) and immunohistochemical (IHC)

TMA chip was purchased from Outdo Biotech, Ltd (HProA060PG01, Shanghai, China), consisting of the prostate tumor tissue specimens (*n* = 49) and adjacent normal prostate tissue specimens (*n* = 11). IHC was executed on a TMA chip using primary antibodies against METTL14 (HPA038002, Sigma). The Image J software was used to quantify the protein levels by calculating the integrated optical density per stained area (IOD/area). The clinical information of the patients was provided in Supplementary Table 1.

### Quantitative PCR (qPCR) assays

Total RNA from tumor cells was extracted with Trizol (15596-026) (Invitrogen, Carlsbad, CA) and reverse transcribed using the HiScript III 1st Strand cDNA Synthesis Kit (R123-01, Vazyme, China). The qRT-PCR assay was performed using ChamQ SYBR Master Mix (Q311-02) (Vazyme, Nanjing, China). Relative expression of mRNA was calculated by normalization to ACTB as the endogenous control. At least three independent replicates were included for analysis. The primers used were listed as follows: METTL14: sense: 5’-GTTGGAACATGGATAGCCGC-3’; antisense: 5’-CAATGCTGTCGGCACTTTCA-3’; THBS1: sense: 5’-AGAATGCTGTCCTCGCTGTT-3’; antisense: 5’-TTTCTTGCAGGCTTTGGTCT-3’; YTHDF2: sense: 5’-AGCCCCACTTCCTACCAGATG-3’; antisense: 5’-TGAGAACTGTTATTTCCCCATGC-3’.

### Western blot

Cells were lysed in RIPA buffer (89901) (Invitrogen, Carlsbad, CA) including protease inhibitor (HY-K0021, MCE, NJ). The protein abundance was measured by BCA Protein Assay Kit (23227) (Invitrogen, Carlsbad, CA). The lysates were centrifuged at 12,000 × *g* for 10 min at 4 °C to remove cell debris. Afterwards, the supernatants were subjected to SDS-PAGE for the following subsequent experiments. The PVDF membranes with transfected protein were incubated at 4 °C overnight and then incubated with secondary antibodies (31460, Invitrogen) for 2 h. Finally, the PVDF membranes were washed using 1% TBST and detected by the Bio-Rad ChemiDoc Touch Imaging System. The antibodies used were as follows: GAPDH (30203ES50, Yeasen, China), METTL14 (HPA038002, Sigma), THBS1 (ab267388, abcam), YTHDF2 (24744-1-AP, proteintech). The original western blots were provided in Supplementary Material.

### Lentivirus package and infection

Plasmids including transgenes and packaging plasmids were co-transfected into HEK 293 T cells using polyethylenimine linear (PEI) (40815ES03, Yeasen, China). After 48 h, viruses were collected. When tumor cells grew at a density of around 50%, the collected viruses were transfected with an appropriate concentration of polybrene (ST1380-10, Beyotime, China). After 2 days, puromycin (58-58-2, Gene Operation, NY) was used to select the transfected cells.

### Immunofluorescence (IF)

Cells were plated on coverslips in 12-well plates and were harvested when growing to an ~40% density. The slides were fixed with formalin for 15 min and washed twice with phosphate buffer saline (PBS). Then, 2 ml 0.2–0.5% triton X-100 was used for permeabilization for 10 min. Blocking solution was used to block the cells for 30 min and then the primary antibody was incubated at room temperature for 1 h. After combination, the slides were washed with PBS for 3 × 5 min. Next, the secondary antibody (1:1000, Invitrogen) was combined for 1 h at room temperature in the darkness and followed by three times of PBS washing. Finally, we added a drop of mounting medium on the slides for 10 min and checked the staining results with a fluorescence microscope.

### Cell proliferation and colony formation assay

For cell proliferation assay, control and transfected tumor cells were seeded into 96-well plates in a density of 1500 cells/well. For every 24 h, replicate wells were added 10% CCK8 (MA0218-3, meilunbio, China) and maintained at 37 °C for 2 h. Subsequently the absorbance was measured at 450 nm for each well.

For colony formation assay, 500 cells per well were seeded into six-well plates and cultured in the medium with 10% FBS in the incubator for 2 weeks. Then, the cells were fixed with 4% paraformaldehyde (AR-0211, FuDing, China) and afterwards stained with crystal violet (C0121, Beyotime, China). Finally, the colonies were measured and counted.

### Cell cycle assay

The control and transfected cells (5 × 10^5^) were collected and then washed with PBS. Accordingly, 1 ml DNA Staining solution and 10 μl permeabilization solution of the cell cycle staining kit (70-CCS012, MultiSciences, China) were added into each sample, and then the samples were left at room temperature for 30 min. After these treatments, cells were measured by flow cytometry, and the data were analyzed by Cell Quest Modfit software.

### Xenografts in mice

Stably transfected shMETTL14 and shNC DU145 cells (5 × 10^6^ cells) suspended in a mixture of 100 μL PBS were subcutaneously injected into the right flank of male nude BALB/C mice (6–8 weeks old) to induce tumor formation. Tumor growth was measured every week using a caliper and the tumor volume was calculated by the formula (width)^2^ × length/2. All animal studies were consistent with the Shanghai Jiaotong University Guide for the care and use of laboratory animals.

### siRNA transfection

Tumor cells were seeded on the six-well plates at a density of 2 × 10^5^ cells per well. After 24 h, cells were transfected with transient knockdown of target genes by siRNA using Lipofectamine 3000 reagent (Invitrogen, Carlsbad, CA). Cells were harvested 48 h for RNA abundance analysis and 72 h for protein expression analysis. YTHDF2 and THBS1 siRNA sequences and negative control sequences were designed and synthesized by Genomeditech (Shanghai, China). Sequences of siRNAs were listed in Supplementary Table 2.

### m6A quantification assay

Total RNA was extracted from prostate cancer cells using Trizol. Extracted 200 ng RNA was used by EpiQuik m6A Methylation Quantification Kit (P9005-48) (Epigentek, Farmingdale, NY), and the m6A level of each sample was measured according to the manufacturer’s instructions.

### RNA immunoprecipitation (RIP) assays

RIP assays were performed using Magna RIP RNA-Binding Protein Immunoprecipitation Kit (17–700) (Millipore, Billerica, MA) according to the manufacturer’s instructions. 2 × 10^7^ DU145 or PC3 cells were collected using Lysis Buffer. Magnetic Beads Protein A/G was incubated with antibodies against YTHDF2 and IgG overnight at 4 °C. The next day after times of washing and other treatments, the purified RNA was conducted by qPCR, and data were normalized to input.

### Methylated RNA immunoprecipitation sequencing (MeRIP-seq) and MeRIP-qPCR

In brief, MeRIP assays were conducted following the manufacturer’s instructions (C11051-1, RiboBio, China). Total 100ug RNA of shMETTL14 and shNC were fragmented into 100–150 bp fragments. Then, about 1/10 of the fragment RNA was divided as input. The others were coated with anti-m6A (ab190886, Abcam) or IgG for 2 h at 4 °C. After times of washing and other procedures, the methylated RNA was purified by Magen Hipure Serum/plasma miRNA Kit (R4317-03, Magen, China) and the samples were sequenced by RiboBio (Guangzhou, China). For MeRIP-qPCR, the purified methylated RNA from the above steps was reverse-transcribed and analyzed by qPCR.

### RNA stability assays

PCa cells were plated in twelve-well plates. The following day, actinomycin D (5 ug/ml, S8964, Selleck) was added into the culture medium. Total RNA was collected at 0, 3, 6 h and analyzed by qPCR. The data were normalized by β-Actin and evaluated with a linear regression model.

### Statistical analysis

Statistical analysis was carried out using the GraphPad Prism software (version 8.0). To compare two independent groups, a two-tailed Student’s *t*-test was used, whereas, for differences among more groups, one-way analysis of variance (ANOVA) was conducted on the data. *p* < 0.05 was considered statistically significant. Survival analysis was performed using Kaplan–Meier methods, and a log-rank test was used to determine the statistical significance of differences.

## Supplementary information

Supplementary Figure 1

Supplementary Table 1

Supplementary Table 2

Supplementary Table 3

Supplementary Table 4

Supplementary Table 5

Supplementary Figure&Table Legends

Original western blots

## Data Availability

All data generated or used during this study are available from the corresponding author by request.

## References

[1] Zhu Y, Mo M, Wei Y, Wu J, Pan J, Freedland SJ (2021). Epidemiology and genomics of prostate cancer in Asian men. Nat Rev Urol.

[2] Kyjacova L, Hubackova S, Krejcikova K, Strauss R, Hanzlikova H, Dzijak R (2015). Radiotherapy-induced plasticity of prostate cancer mobilizes stem-like non-adherent, Erk signaling-dependent cells. Cell Death Differ.

[3] Rossini A, Giussani M, Ripamonti F, Aiello P, Regondi V, Balsari A (2020). Combined targeting of EGFR and HER2 against prostate cancer stem cells. Cancer Biol Ther.

[4] Huang Y, Jiang X, Liang X, Jiang G (2018). Molecular and cellular mechanisms of castration resistant prostate cancer. Oncol Lett.

[5] Chen M, Wei L, Law CT, Tsang FH, Shen J, Cheng CL (2018). RNA N6-methyladenosine methyltransferase-like 3 promotes liver cancer progression through YTHDF2-dependent posttranscriptional silencing of SOCS2. Hepatology.

[6] Kumaraswamy A, Welker Leng KR, Westbrook TC, Yates JA, Zhao SG, Evans CP (2021). Recent advances in epigenetic biomarkers and epigenetic targeting in prostate cancer. Eur Urol.

[7] Lewis CJ, Pan T, Kalsotra A (2017). RNA modifications and structures cooperate to guide RNA-protein interactions. Nat Rev Mol Cell Biol.

[8] Fu Y, Dominissini D, Rechavi G, He C (2014). Gene expression regulation mediated through reversible m^6^A RNA methylation. Nat Rev Genet.

[9] Ramalingam H, Kashyap S, Cobo-Stark P, Flaten A, Chang CM, Hajarnis S (2021). A methionine-Mettl3-N(6)-methyladenosine axis promotes polycystic kidney disease. Cell Metab.

[10] Liu X, Wang H, Zhao X, Luo Q, Wang Q, Tan K (2021). Arginine methylation of METTL14 promotes RNA N(6)-methyladenosine modification and endoderm differentiation of mouse embryonic stem cells. Nat Commun.

[11] Sun HL, Zhu AC, Gao Y, Terajima H, Fei Q, Liu S (2020). Stabilization of ERK-phosphorylated METTL3 by USP5 increases m(6)A methylation. Mol Cell.

[12] Yang Y, Hsu PJ, Chen YS, Yang YG (2018). Dynamic transcriptomic m(6)A decoration: writers, erasers, readers and functions in RNA metabolism. Cell Res.

[13] Lin S, Choe J, Du P, Triboulet R, Gregory RI (2016). The m(6)A methyltransferase METTL3 promotes translation in human cancer cells. Mol Cell.

[14] Yao QJ, Sang L, Lin M, Yin X, Dong W, Gong Y (2018). Mettl3-Mettl14 methyltransferase complex regulates the quiescence of adult hematopoietic stem cells. Cell Res.

[15] Dong L, Chen C, Zhang Y, Guo P, Wang Z, Li J (2021). The loss of RNA N(6)-adenosine methyltransferase Mettl14 in tumor-associated macrophages promotes CD8(+) T cell dysfunction and tumor growth. Cancer Cell.

[16] Chen Y, Pan C, Wang X, Xu D, Ma Y, Hu J (2021). Silencing of METTL3 effectively hinders invasion and metastasis of prostate cancer cells. Theranostics.

[17] Du L, Li Y, Kang M, Feng M, Ren Y, Dai H (2021). USP48 is upregulated by Mettl14 to attenuate hepatocellular carcinoma via regulating SIRT6 stabilization. Cancer Res.

[18] Chen X, Xu M, Xu X, Zeng K, Liu X, Pan B (2020). METTL14-mediated N6-methyladenosine modification of SOX4 mRNA inhibits tumor metastasis in colorectal cancer. Mol Cancer.

[19] Dong L, Chen C, Zhang Y, Guo P, Wang Z, Li J (2021). The loss of RNA N(6)-adenosine methyltransferase Mettl14 in tumor-associated macrophages promotes CD8(+) T cell dysfunction and tumor growth. Cancer Cell.

[20] Choi SH, Tamura K, Khajuria RK, Bhere D, Nesterenko I, Lawler J (2015). Antiangiogenic variant of TSP-1 targets tumor cells in glioblastomas. Mol Ther.

[21] Beretta G, Moretti RM, Nasti R, Cincinelli R, Dallavalle S, Montagnani Marelli M (2021). Apoptosis-mediated anticancer activity in prostate cancer cells of a chestnut honey (Castanea sativa L.) quinoline-pyrrolidine gamma-lactam alkaloid. Amino Acids.

[22] Panneerdoss S, Eedunuri VK, Yadav P, Timilsina S, Rajamanickam S, Viswanadhapalli S (2018). Cross-talk among writers, readers, and erasers of m(6)A regulates cancer growth and progression. Sci Adv.

[23] Zhao W, Qi X, Liu L, Ma S, Liu J, Wu J (2020). Epigenetic regulation of m(6)A modifications in human cancer. Mol Ther Nucleic Acids.

[24] Shi H, Wei J, He C (2019). Where, when, and how: context-dependent functions of RNA methylation writers, readers, and erasers. Mol Cell.

[25] Wang X, Lu Z, Gomez A, Hon GC, Yue Y, Han D (2014). N6-methyladenosine-dependent regulation of messenger RNA stability. Nature.

[26] Meyer KD, Saletore Y, Zumbo P, Elemento O, Mason CE, Jaffrey SR (2012). Comprehensive analysis of mRNA methylation reveals enrichment in 3’ UTRs and near stop codons. Cell.

[27] Wang M, Liu J, Zhao Y, He R, Xu X, Guo X (2020). Upregulation of METTL14 mediates the elevation of PERP mRNA N(6) adenosine methylation promoting the growth and metastasis of pancreatic cancer. Mol Cancer.

[28] Yang Z, Yang S, Cui YH, Wei J, Shah P, Park G (2021). METTL14 facilitates global genome repair and suppresses skin tumorigenesis. Proc Natl Acad Sci USA.

[29] Li J, Xie H, Ying Y, Chen H, Yan H, He L (2020). YTHDF2 mediates the mRNA degradation of the tumor suppressors to induce AKT phosphorylation in N6-methyladenosine-dependent way in prostate cancer. Mol Cancer.

[30] Jiménez B, Volpert OV, Crawford SE, Febbraio M, Silverstein RL, Bouck N (2000). Signals leading to apoptosis-dependent inhibition of neovascularization by thrombospondin-1. Nat Med.

[31] Moserle L, Jiménez-Valerio G, Casanovas O (2014). Antiangiogenic therapies: going beyond their limits. Cancer Discov.

[32] Yang HD, Kim HS, Kim SY, Na MJ, Yang G, Eun JW (2019). HDAC6 suppresses Let-7i-5p to Elicit TSP1/CD47-mediated anti-tumorigenesis and phagocytosis of hepatocellular carcinoma. Hepatology.

[33] Zhang Y, Zheng D, Zhou T, Song H, Hulsurkar M, Su N (2018). Androgen deprivation promotes neuroendocrine differentiation and angiogenesis through CREB-EZH2-TSP1 pathway in prostate cancers. Nat Commun.

[34] Wang H, Zuo H, Liu J, Wen F, Gao Y, Zhu X (2018). Loss of YTHDF2-mediated m(6)A-dependent mRNA clearance facilitates hematopoietic stem cell regeneration. Cell Res.

[35] Liu J, Lichtenberg T, Hoadley KA, Poisson LM, Lazar AJ, Cherniack AD (2018). An integrated TCGA pan-cancer clinical data resource to drive high-quality survival outcome analytics. Cell.

