# Supplementary figures and images for: METTL14 promotes prostate tumorigenesis by inhibiting THBS1 via an m6A-YTHDF2-dependent mechanism
Source: Cell Death Discov. 2022 Mar 30;8:143. doi: 10.1038/s41420-022-00939-0 (PMC8967870; doi:10.1038/s41420-022-00939-0)

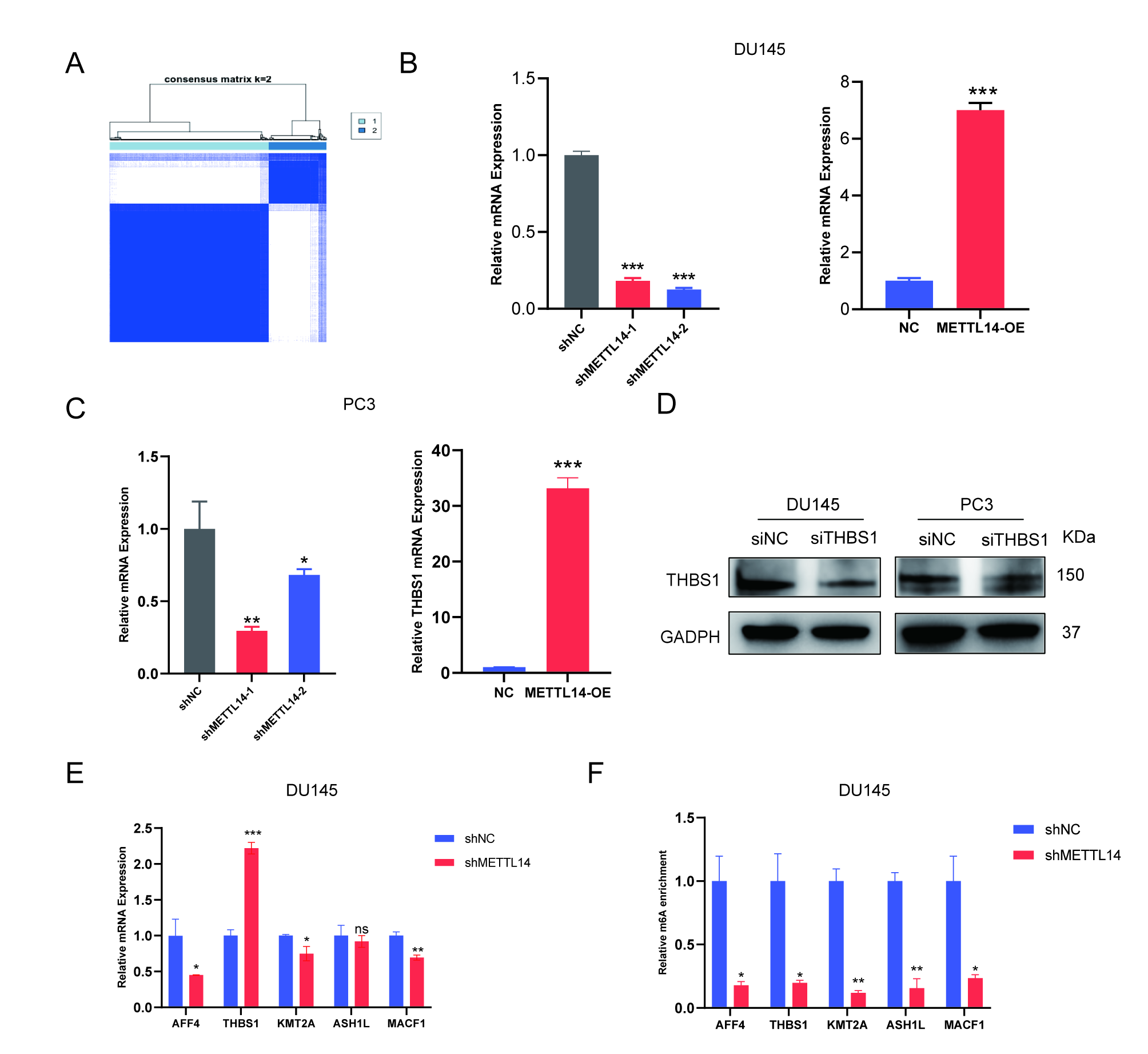

Supplement: Supplementary file 1 — Supplementary Figure 1 [file 41420_2022_939_MOESM1_ESM.tif]

Figure 1D

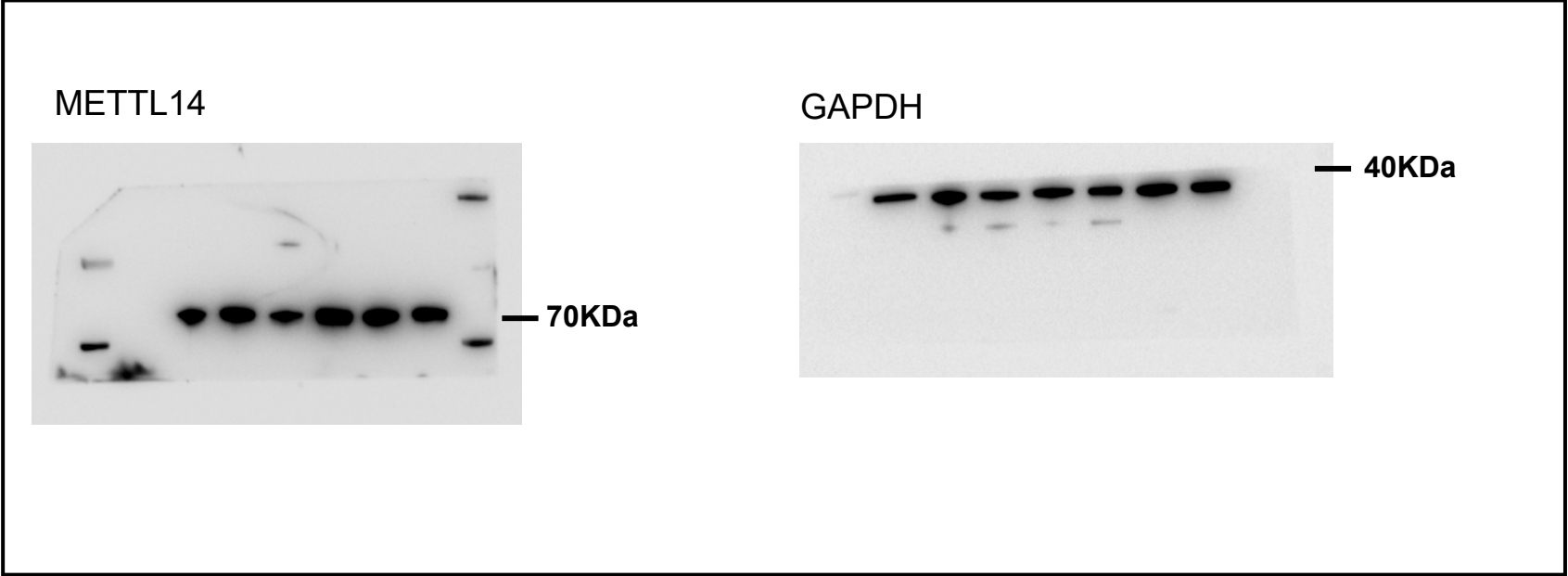

Figure 2A

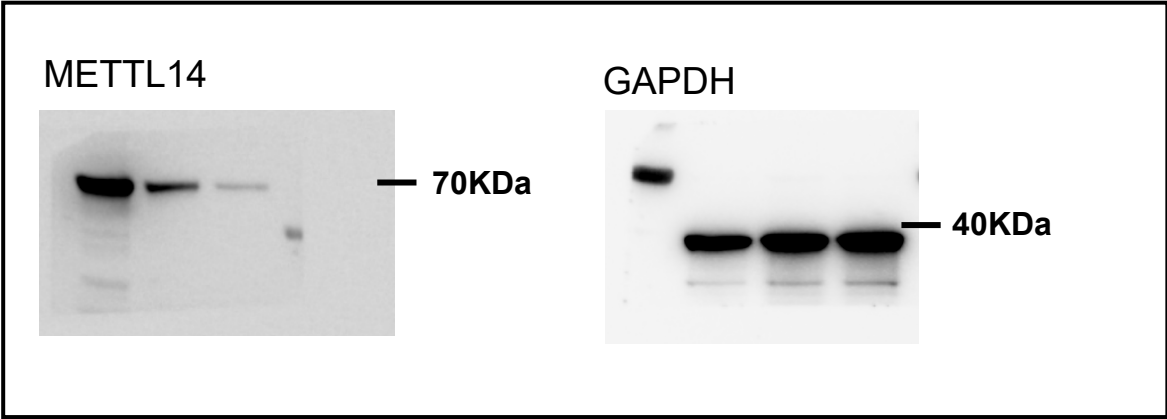

Figure 2D

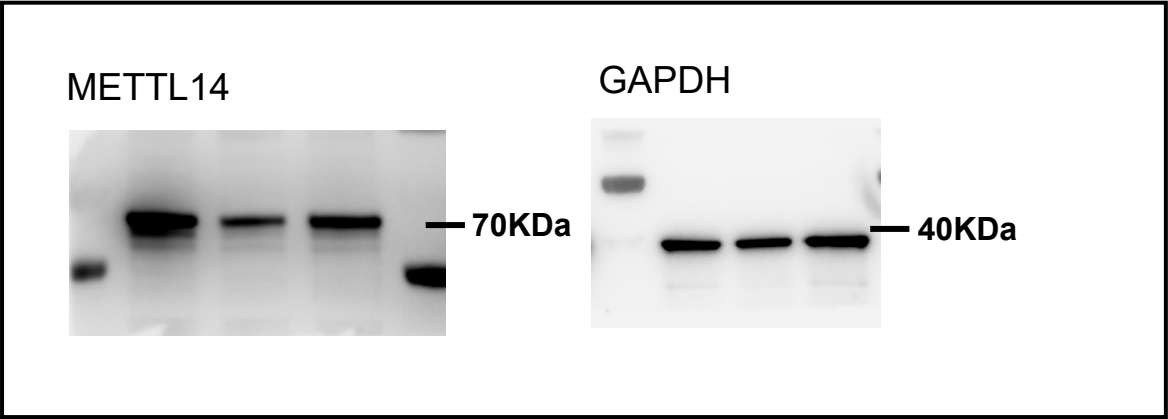

Figure 2L

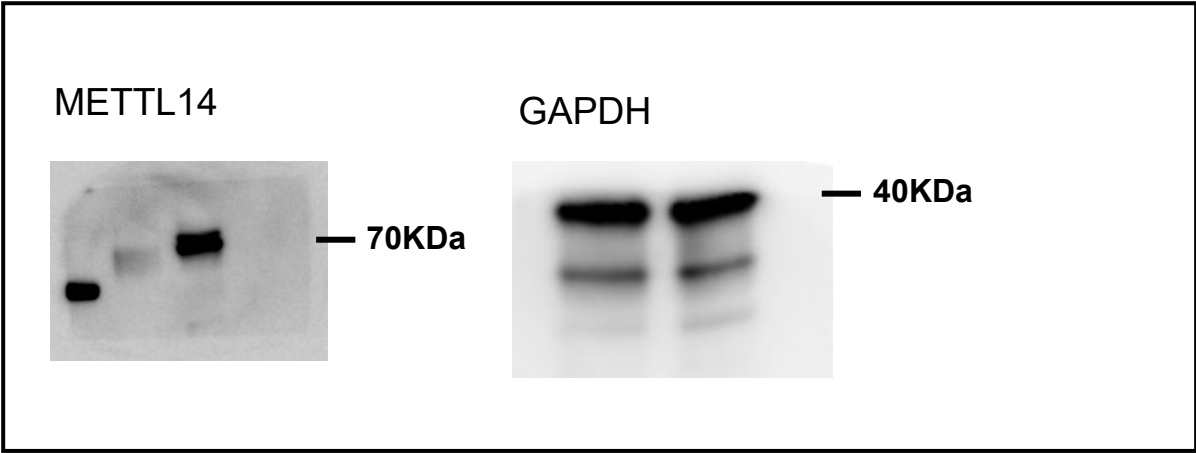

Figure 4D

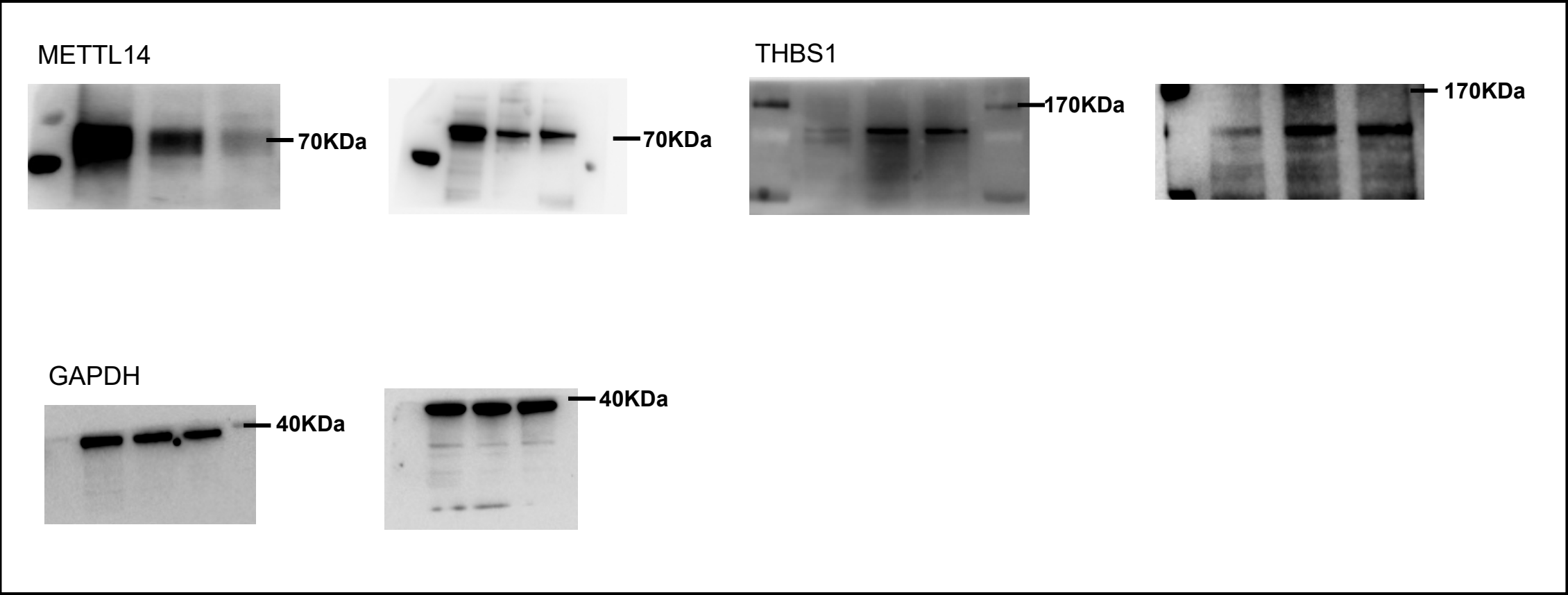

Figure 5D, 5F

YTHDF2

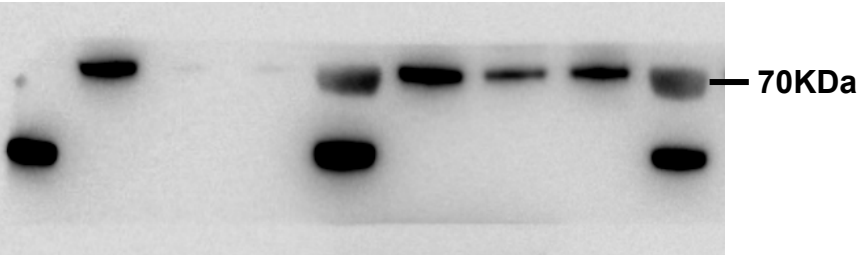

THBS1

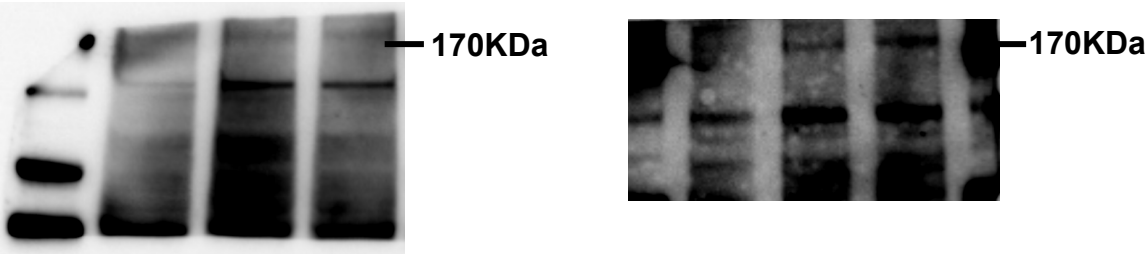

GAPDH

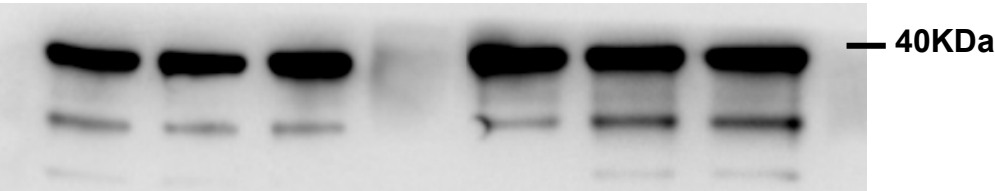

Supplementary Figure 1D

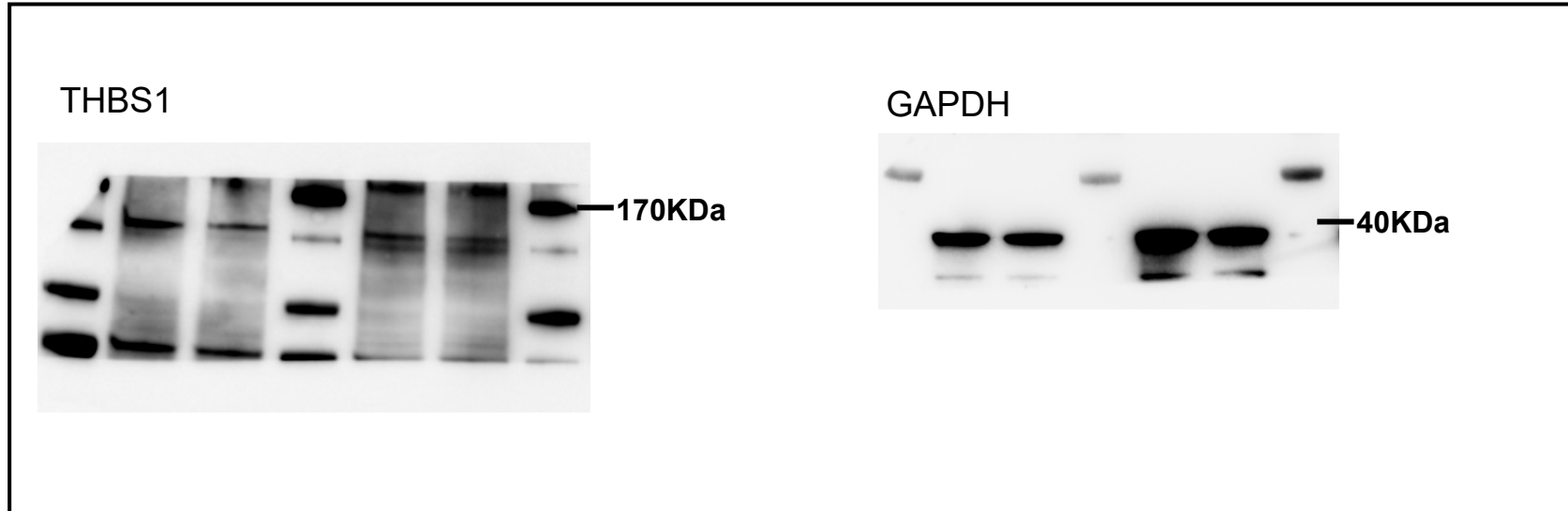

Supplement: Supplementary file 8 — Original western blots [file 41420_2022_939_MOESM8_ESM.pdf]
