# Supplementary material for: METTL14 promotes prostate tumorigenesis by inhibiting THBS1 via an m6A-YTHDF2-dependent mechanism
Source: Cell Death Discov. 2022 Mar 30;8:143. doi: 10.1038/s41420-022-00939-0 (PMC8967870; doi:10.1038/s41420-022-00939-0)
Supplement: Supplementary file 7 — Supplementary Figure&Table Legends [file 41420_2022_939_MOESM7_ESM.docx]

**Supplementary Fig. 1**

**A** Heatmap of Consensus matrix for k=2 based on consensus clustering of m6A gene expression. **B, C** The knockdown and overexpression efficiency of METTL14 in DU145 **(B)** and PC3 **(C)** cells. **D** The knockdown efficiency of THBS1 using targeted siRNA. **E** mRNA level of shared genes of RNA-seq and MeRIP-seq in DU145 shNC and shMETTL14 cells. **F** Relative m6A enrichment of shared genes in DU145 shNC and shMETTL14 cells. All data are presented as the mean ± SDs. * p<0.05; ** p<0.01; *** p<0.001 (Student’s t-test).

**Supplementary Table 1**:

The clinical data of the patients in human prostate cancer tissue microarrays.

**Supplementary Table 2**:

siRNA sequences used in this study.

**Supplementary Table 3**:

Differential genes of RNA-seq in shNC and shMETTL14 in DU145 cells.

**Supplementary Table 4**:

GO analysis of differential genes of RNA-seq.

**Supplementary Table 5**:

Reduced m6A peaks of MeRIP-seq of shNC and shMETTL14 DU145 cells.
